# The development and validation of a disease-specific quality of life measure in hyperhidrosis: the Hyperhidrosis Quality of Life Index (HidroQOL©)

**DOI:** 10.1007/s11136-014-0825-2

**Published:** 2014-11-01

**Authors:** P. Kamudoni, B. Mueller, M. S. Salek

**Affiliations:** 1 Centre for Socioeconomic Research, School of Pharmacy and Pharmaceutical Sciences, Cardiff University, Redwood Building, Kind Edward VII Avenue, Cardiff, CF 10 3NB UK; 2 Medical Science and Operations Department, Riemser Pharma GmbH, Greifswald, Germany

**Keywords:** Patient-reported outcome measure, Quality of life, Hyperhidrosis, Excessive sweating, Hyperhidrosis Quality of Life Index, HidroQoL

## Abstract

**Purpose:**

To develop and validate a new disease-specific quality of life measure in hyperhidrosis for use in both routine clinical practice and clinical research.

**Methods:**

Interviews and focus group discussions with hyperhidrosis patients, reported elsewhere, provided the content for the measure validated in this study (*n* = 71). A panel of dermatologists (*n* = 5) and patients (*n* = 7) carried out content validation. Further, item reduction and the initial construct validation were carried out in a cross-sectional study (*n* = 595), using the unidimensional Rasch analysis and exploratory factor analysis. Subsequently, the construct validity, reliability and responsiveness of the revised measure were assessed in a longitudinal study (*n* = 260). Data collection for the item reduction and the final validation phases was entirely carried out online.

**Results:**

The expert panels judged the HidroQoL as content valid. Rasch analysis supported the revision of response options from five to three. Following removal of misfitting items, a set of 15 items showed optimal fit to the model (chi-squared statistic = 159.64, *p* = 0.07). Three additional items were retained on consideration of their importance to patients, resulting in an 18-item instrument. The items were grouped into two subscales, *daily life activities* and *psychosocial life* domains, based on results of the factor analysis. In subsequent construct validation, the HidroQoL correlated with the DLQI (*r*_s_ = 0.6, *p* < 0.01). Reliability was high (internal consistency, Cronbach’s alpha: overall scale = 0.9; test–retest reliability, Intra-class correlation = 0.9). The HidroQoL scores were sensitive to change in patients’ disease severity (score change from baseline to follow-up after 15–35 days, Cohen’s ES = 0.47).

**Conclusion:**

This study has provided the initial evidence supporting measurement properties and the use of the HidroQoL instrument in both routine clinical practice and in research, for assessing quality of life impacts in hyperhidrosis.

**Electronic supplementary material:**

The online version of this article (doi:10.1007/s11136-014-0825-2) contains supplementary material, which is available to authorized users.

## Introduction

Hyperhidrosis, a skin disorder characterised by excessive sweating without aetiology [1], results in substantial impairment in patients’ daily life [2, 3]. Assessing such impacts is key to confirming the diagnosis and establishing the severity of the condition [4], given the difficulty of quantifying and interpreting laboratory-based measurements of disease severity in hyperhidrosis [5]. The measurement of health-related quality of life (HRQoL), therefore, is central to the clinical management of hyperhidrosis, suggesting the need for measures that are appropriate and fit for purpose. Psychometric attributes such as validity (that an instrument indeed measures what it purports to measure [6]) and reliability (that scales are internally consistent and yield reproducible scores) are an important consideration in determining this. For instruments used longitudinally, responsiveness, a measure’s ability to detect small but clinically important change over time [7], is also required. Ultimately, the usefulness of a measure depends on the interpretability of its scores, the ability to decipher clinically relevant meaning from the scores [8].

The measures currently in use for assessing HRQoL in hyperhidrosis were reviewed. Generic HRQoL measures such as the SF-36 or the NHP consist of items irrelevant for hyperhidrosis patients, while omitting some key issues [9]. This also applies to dermatology-specific measures (Skindex, Dermatology Life Quality Index-DLQI, and Patient Benefit Index) albeit to a lesser degree. Among the disease-specific measures, including those where patient involvement in the development process (concept elicitation) was mentioned (Hyperhidrosis Impact Questionnaire—HHIQ; Hyperhidrosis Questionnaire—HQ; Amir’s Quality of Life Instrument), it was not possible to establish that the content was appropriate and had the right emphasis for patients with hyperhidrosis. It was also unclear whether the experience and views of hyperhidrosis patients in Israel (for the ‘Amir’s instrument) and Korea (for the HQ) would be similar/relevant/comparable with UK or US patients.

Only one out of five disease-specific measures (HHIQ) provided information on all basic psychometric properties (reproducibility, construct/convergence validity/external validity and responsiveness). Among the other measures, the assessment or reporting of psychometric properties such as reliability and construct validity was often poor [10]. None of the disease-specific instruments has been evaluated based on modern test theory, for example, differential item functioning for key demographic factors has not been assessed. Clinical appropriateness was largely overlooked. Even the most promising measure, the HHIQ, has not been adapted for use in routine clinical practice [11].

It is considered to be a good practice to build upon existing measures (e.g. improved or shortened) instead of developing a new measure to overcome the inadequacies of the existing ones. In the first instance, the onus of such approach should be on the original developers. Other researchers making such an attempt would be faced with the difficulty of not having access to the original data, opposition of the original developers being protective about their measure and attitude of the scientific journals towards publishing such work. Such instrument modification work stands to be considered a compromise to starting on a clean slate without any background noise or bias that could be inherent in the existing measures.

There is therefore an urgent need for a fully validated pragmatic instrument for evaluating hyperhidrosis-specific QoL, for use in both routine clinical practice and clinical research. Such an instrument could enhance the diagnosis and management of the condition. Also, a practical measure may make it easy to integrate HRQoL information into discussions between clinicians and patients during consultation [12]. The current study therefore describes the development and validation of such an instrument, the Hyperhidrosis Quality of Life Index (HidroQoL©).

### Development of the new instrument

The new instrument was developed based on interviews and focus groups carried out to investigate the impacts of hyperhidrosis on patients’ lives (*n* = 71) [13]. A manuscript containing the results of the qualitative study is currently undergoing submission. The themes and subthemes identified from the study were used in developing the conceptual framework and items of the new measure, based on the following criteria: all issues with prevalence of ≥5 % were included; item phrasing was based on the language used by patients and at the reading level of a 12-year old and item stems were suitable for and consistent with the response categories [14]. The resultant prototype instrument contained 47 items scored on a 6 point scale (No not at all, A little, Somewhat, Quite a bit, Very much and Not relevant).

## Methods

### Ethical approval and patient consent

This study was approved by the ethics committee of the University Hospital of Greifswald, Germany. As the data collection was carried out online and was based in Greifswald/Germany, the local ethics committee (S Wales) waived the need for ethical clearance in Wales/UK. All participants gave written informed consent prior to their participation in the study.

### Patient population

The patients participating in this study (Steps 2 and 3) were recruited through online social networking communities for hyperhidrosis, mostly drawn from the International Hyperhidrosis Society and the UK Hyperhidrosis Support Group. Both groups maintain an internet portal, a Facebook group/page and circulate an e-mail-based newsletter periodically, for sharing information among members. An advert about the study containing contact details of the research team and a link to the study website was posted across all online communication channels of the groups. Patients who contacted the research team, fulfilling the inclusion/exclusion criteria and willing to give written informed consent were subsequently recruited into the study. Study participants had self-reported hyperhidrosis, were aged 18 years or above, had a Hyperhidrosis Disease Severity Score (HDSS) of at least 2 (*tolerable sweating but sometimes interferes with daily activities*), and onset of hyperhidrosis at or before early adult years. There were no incentives offered to patients for their participation in the study.

### Study design

A mixed methods design with multiple steps was followed in this study. In Step 1, content validation was assessed by two expert panels (patients, *n* = 7 and clinicians, *n* = 5), quantitatively using a questionnaire (*content validation questionnaire*) administered by e-mail and through an expert panel discussion. Recommendations provided by panel members on any aspects of the HidroQoL were documented. In Step 2, initial construct validation and item reduction were carried out based on patient responses to the developmental instrument in a cross-sectional study (cohort 1, *n* = 595). In Step 3, further validation was carried out on the new instrument (final version) to establish reliability, construct validity and responsiveness. This involved implementing a longitudinal study, with patients assessed on three occasions using the final version of the new instrument: at baseline, after 7 and after 21 days (cohort 2, *n* = 260). Reliability was tested by assessing internal consistency (using the baseline assessment) and test–retest reliability (assessed by examining reproducibility of scores from baseline to first follow-up in those with a stable condition). Construct validity of the HidroQoL was tested by evaluating its relationship with other measures of disease activity and disease impact in hyperhidrosis. Responsiveness was tested in a longitudinal study, by assessing change in scores from baseline to second follow-up after 15–35 days.

### Measures

In the content validation questionnaire, used in step 1, each item was rated for language clarity, completeness, relevance and appropriateness of response scaling, according to a 4 point Likert scale (1 = strongly disagree, 2 = disagree, 3 = agree, 4 = strongly agree). During Steps 2 and 3, patients completed the following instruments, in addition to the new measure under development: the Hyperhidrosis Disease Severity Scale (HDSS), a measure of self-assessed disease severity and daily life interference in hyperhidrosis [15]; the Dermatology Life Quality Index (DLQI) [16–18] and the Skindex-17 [19, 20], measures of skin-related QoL. The overall impact of hyperhidrosis on the patient was assessed using the General Question (GQ): *Over the last 7* *days including today, how much has your sweating condition affected your life?* A question with similar phrasing has been previously used in instrument validation work in dermatology and in renal failure [17, 21]. The burden of hyperhidrosis was also assessed in terms of daily time spent in managing the condition, as in a previous work in atopic dermatitis [22]. Data were also collected on socio-demographic and disease characteristics including *co*-*morbidities*, *body site affected and treatment history.*

### Procedure

A web version of the new instrument was developed and made accessible through a purposively developed website for the study. The landing page of the site provided basic study information, with additional patient-related information (e.g. a downloadable full patient information sheet) placed elsewhere on the site. Access to the questionnaire area required a valid e-mail address, patient consent and a password. Consent was provided electronically. Information about the study was posted on various online social networking communities/sites related to hyperhidrosis.

### Data processing and analysis

Data analyses in the initial construct validation step, involving exploratory factor analysis (EFA) and Rasch analysis, were carried out using M-PLUS 6 and RUMM2030, respectively. In implementing the EFA, the Weighted Least Squares (WLSMV) estimator and the Geomin routine were used for factor estimation and for the subsequent factor rotation [23]. The optimal number of factors was identified using Cattel’s scree plot and Horns parallel analysis [24]. Candidate items for removal had their highest factor loading ≤0.4, a loading of 0.4–0.5 on more than a single factor, residual variance ≥0.7, or poor content match with their dominant factor [25].

In the Rasch analysis, model fit was assessed for the entire scale, the individual items and the persons. Optimal overall model fit is shown by *mean fit residuals* of 0, a standard deviation of 1–1.5 and a non-significant (*p* > 0.05) total item–trait interaction chi-squared statistic [26]. Fit residuals <|±2.5| indicate optimal fit for the individual items/persons [27]. Unidimensionality and local dependence assumptions were assessed by exploring patterns in the residuals after fitting the Rasch model [28]. Differential item functioning across patient characteristics was assessed using a two-way ANOVA test. A significant main effect for a demographic variable indicates the presence of uniform DIF, while a significant interaction effect (demographic variable × underlying impairment in QoL) indicates the presence of non-uniform DIF [29].

The rest of the data analysis was carried out using SPSS. Internal consistency of scales was measured using Cronbach’s alpha and corrected item–total correlations. Test–retest reliability was assessed by measuring the level of agreement between baseline and first follow-up score using intra-class correlations. The relationship between the HidroQoL and other measures, to establish construct validity, was assessed using spearman’s rank-sum correlations. A correlation of 0–0.09 is considered poor, 0.1–0.2 slight, 0.21–4 fair, 0.41–0.6 moderate, 0.61–0.8 substantial and 0.81–1 is considered perfect [30]. To assess responsiveness, the change in score between baseline and third assessment was measured using a paired *t* test. Magnitude of change was captured using Cohen’s effect size. The relative precision of the new measure in detecting change was estimated as a ratio of the *t* test statistics for the new measure versus that obtained for the DLQI.

## Results

### Content validity

The HidroQoL was rated content valid by the expert panels. The data collected allowed revision of the instrument. The recall period was changed from ‘*at present*’ to ‘*the last 7* *days including today,*’ and the option ‘*not relevant*’ was removed from the response options. One item was deleted, twenty-nine were revised, and three were added, resulting in a 49-item developmental instrument, scored on a 5-point Likert scale.

### Construct validation and item reduction

The characteristics of the patients participating in all phases of the study are reported in Table 1. Correlation analysis (based on USA patients from cohort 1, *n* = 559) showed 30 items with *polychoric correlation coefficient* >0.8, suggesting multicollinearity. Following consideration of content overlap and importance of the issues to patients (based on the results of qualitative study reported by Kamudoni et al. [13]), 13 items were removed, retaining 36 items for subsequent analyses.

**Table 1:** Socio-demographic characteristics of study participants

	Cohort 1 (*n* = 595)	Cohort 2 (*n* = 260)
Gender, *n* (%)		
Male	113 (19 %)	65 (25 %)
Female	482 (81 %)	195 (75 %)
Age, years		
Mean (SD)	40.5 (14.2)	37 (14)
Median	39	33
Range	18–74	17–74
Duration of condition, years		
Mean (SD)	27.5 (14.1)	Na.
Median	25	Na.
Range	2–69	Na.
Body are affected		
Head^a^	129 (22 %)	24 (9 %)
Axilla^a^	54 (9 %)	28 (11 %)
General	130 (22 %)	56 (22 %)
Axilla, palms, feet	158 (27 %)	73 (28 %)
Palms and feet	124 (21 %)	79 (30 %)
Co-morbidity^b^		
Menopausal complaints	61 (10 %)	16 (6.2 %)
Diabetes	30 (5 %)	11 (4.2 %)
Hypertension	47 (8 %)	29 (11.2 %)
Neurological disorders	64 (11 %)	30 (11.5 %)
Thyroid disorders	66 (11 %)	13 (5 %)
Employment status		
Employed	380 (64 %)	160 (61.5 %)
Unemployed	107 (18 %)	37 (14.2 %)
Retired	70 (12 %)	21 (8.1 %)
Full-time student	30 (6 %)	42 (16.2 %)
Country		
USA	559	142 (54.6 %)
Canada	36	11 (4.2 %)
Australia	Na.	11 (4.2 %)
UK	Na.	73 (28.1 %)
Other countries	Na.	23 (9 %)

^a^The classification of the body site of hyperhidrosis reflects the predominant area of sweating

^b^Patients were asked to choose from a list of six conditions including other, to complete statement “I have problems with…”

*Na* not available

#### Exploratory factor analysis

An exploratory factor analysis (EFA) of the HidroQoL (36 items) (based on USA patients from cohort 1, *n* = 559) identified three factors. Twenty-eight items showed clear strong loadings to a single factor, six were cross-loading on several factors, and two had no factor loading >0.4. Sequentially, items with poor performance were removed, with further EFA iteratively carried out, at each step. Subsequently, 21 items loading to two interpretable factors, *daily life activities* and *psychosocial life domain* were retained (Online SM 1). Two factors were to the left of the elbow in the scree plot curve. The factor loadings ranged from 0.53 to 0.89 and 0.58 to 0.94, respectively (Table 2).

**Table 2:** Factor and structure matrix of the HidroQoL (21 items) showing item loadings of the final 21 items fitting the exploratory factor analysis (EFA)

Item	Daily life activities		Psychosocial life		Residual variance
	DLA	SE	PS	SE	Variance
My physical activities are affected	**0.89**	0.02	0.00	0.00	0.20
My everyday housework is affected	**0.83**	0.04	−0.14	0.05	0.44
My hobbies are affected	**0.63**	0.04	0.13	0.05	0.48
I worry about the additional chores in dealing with my condition	**0.59**	0.05	0.24	0.06	0.41
My holidays are affected	**0.56**	0.04	0.29	0.04	0.39
I worry about the additional money in dealing with my condition	**0.53**	0.05	0.22	0.06	0.53
Sweating is constantly on my mind	0.18	0.05	**0.65**	0.04	0.41
My career decisions are affected (e.g. career choice)	0.17	0.05	**0.58**	0.04	0.51
I avoid taking on new challenges	0.13	0.04	**0.76**	0.03	0.29
I avoid public speaking (e.g. presentations)	0.08	0.05	**0.66**	0.05	0.49
My personal relationship are affected	0.08	0.04	**0.73**	0.04	0.39
I feel frustrated	0.03	0.05	**0.78**	0.04	0.36
I feel uncomfortable physically expressing affection (e.g. hugging others)	0.02	0.05	**0.77**	0.04	0.39
I feel sad	0.01	0.04	**0.84**	0.03	0.29
I feel embarrassed	0.00	0.05	**0.86**	0.03	0.25
I find it hard to be near others	0.00	0.03	**0.87**	0.02	0.24
I do not socialise as much as I would like to	0.00	0.03	**0.87**	0.03	0.25
My self-confidence is affected	−0.07	0.04	**0.93**	0.03	0.22
I feel nervous	−0.08	0.04	**0.90**	0.03	0.28
I avoid meeting new people	−0.12	0.04	**0.93**	0.03	0.25
I worry about peoples reaction	−0.17	0.05	**0.94**	0.03	0.30

Factor loadings based on factor pattern matrix represent unique variance in the items attributed to a particular factor

*DLA* daily life activities; *SE* standard error, *PS* psychosocial life domain

The highest factor loading for each item is indicated in bold

#### Rasch analysis

**Fig. 1:**
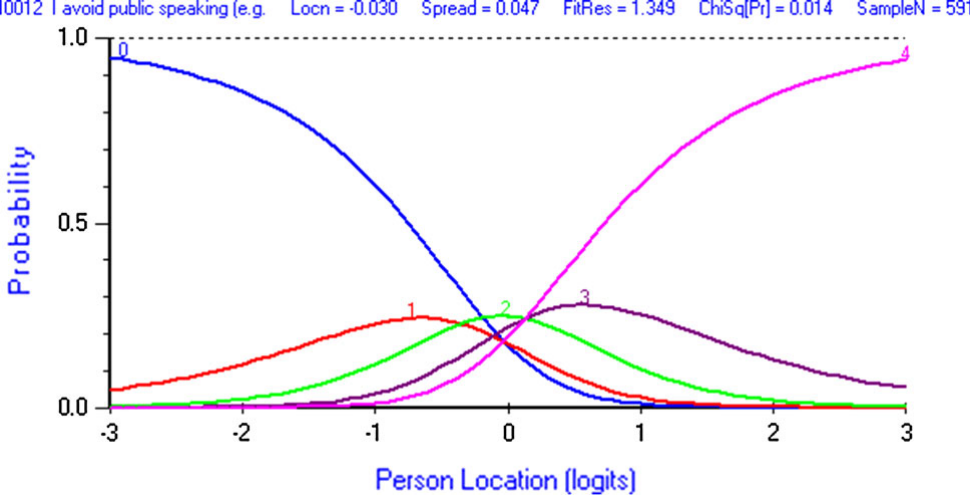
Disordered category thresholds for the item *I avoid public speaking*. Category threshold for scores 0–1 is on a higher location (QoL impairment) than for scores 1–2. Scores 1 (= a little) and 3 (= quite a bit) have no range on the latent QoL variable over which they are most likely

**Fig. 2:**
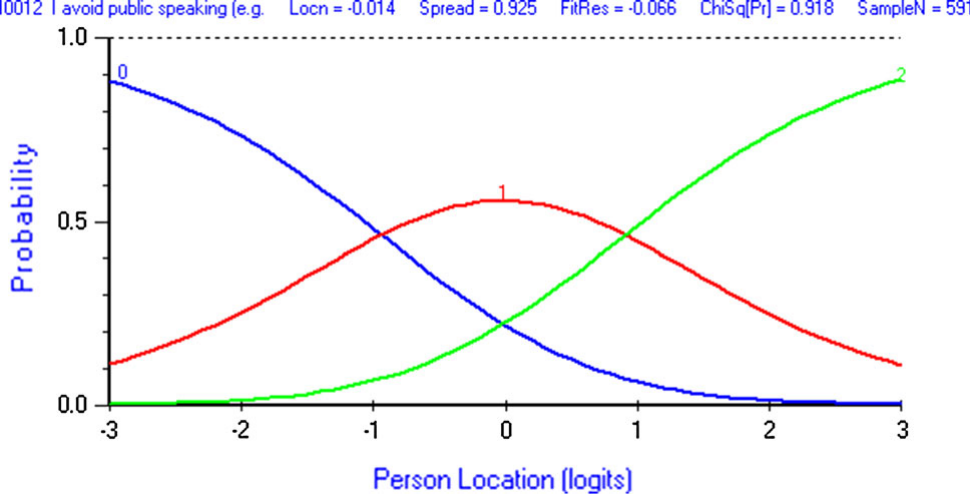
Appropriately ordered category thresholds for the item *I avoid public speaking * after rescoring. Following rescoring from a 5 to a 3 point scale, the category thresholds 0–1 and 1–2 are monotonically ordered. Each score has a range on the latent QoL variable (location) over which it is the most likely

In the Rasch analysis (based on patients from USA and Canada from cohort 1, *n* = 595), the HidroQoL (36 items) showed poor overall fit to the model (total chi-square statistic = 1,642.32, *p* < 0.01), suggesting that it was not unidimensional and that the hierarchical ordering of items according to the underlying HRQoL varied according to its severity. Sixteen items showed good fit (fit residuals <|2.5|), ten items underfitted (fit residuals >2.5), and another ten overfitted (fit residuals <2.5). Three items had optimally functioning response option categories (33 items had disordered category thresholds). A revision of the response option categories from a 5-point to a 3-point scale resolved the dysfunction (Figs. 1, 2).

Misfitting items and those showing local dependence were sequentially removed, retaining a set of 15 items which fulfilled strict unidimensionality requirements [the proportion of pairs of person estimates from two subsets of the HidroQoL items that were significantly different—3.45 % (95 % CI 1.98, 4.92 %)] (Table 3). Six items showed non-uniform DIF for body area, one item for disease severity, and another for co-morbidity. In four items, the observed DIF was revealed to be compensatory rather than real following purification process. Nonetheless, the observed DIF had marginal impact on the performance of the overall scale. Group-specific test characteristic curves (TCC) were near identical for all demographic characteristic (largest difference <0.5 logits) (Online SM 2–5). Therefore, none of the items were removed due to DIF.

**Table 3:** Item fit statistics of the developmental HidroQoL© (15 items) following item reduction with the Rasch model

Item	Location	SE	FitResid	chi-square	p*
My holidays are affected (e.g. planning, activities)	0.51	0.085	−0.51	8.96	0.44
My hobbies are affected	0.02	0.085	1.72	8.62	0.47
I avoid public speaking (e.g. presentations)	0.39	0.077	1.15	16.71	0.05
My work is affected	0.54	0.085	0.74	11.44	0.25
I avoid meeting new people	1.20	0.083	−0.31	5.62	0.78
I feel nervous	−0.72	0.091	−1.99	16.91	0.05
I feel frustrated	−1.03	0.095	−2.30	14.41	0.11
Sweating is constantly on my mind	−1.05	0.096	−1.08	7.59	0.58
My appearance is affected	−0.41	0.088	0.93	8.82	0.45
I worry about leaving sweat marks on things	−1.08	0.096	0.76	9.79	0.37
I worry about people’s reactions	−2.02	0.103	−0.84	14.21	0.11
I feel uncomfortable physically expressing affection (e.g. hugging others)	0.01	0.088	−1.07	9.62	0.38
My sex life is affected	1.62	0.079	0.87	11.17	0.26
I worry about the additional chores in dealing with my condition	1.81	0.086	0.05	7.92	0.54
I find it hard to do things without planning in advance	0.22	0.083	0.54	7.85	0.55

Location indicates the relative level of QoL impairment that each item is targeting, arranging from low (negative) to high impairment (positive)

*p, *p* value; *FitResid*, fit residual

### The final version of the HidroQoL

**Fig. 3:**
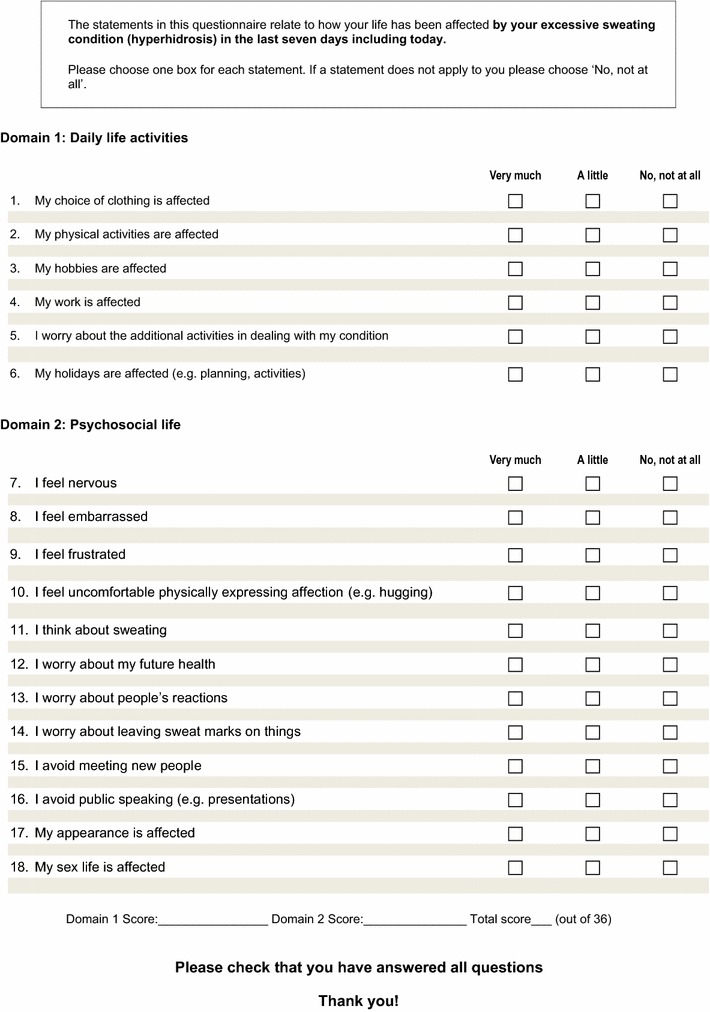
The final version of the HidroQoL© with 18 items. The new hyperhidrosis-specific QoL instrument, the Hyperhidrosis Quality of Life Index

The final version of the HidroQoL adopted all items retained in the Rasch analysis. Three additional items, *my choice of clothing is affected*, *I feel embarrassed* and *my hobbies are affected*, considered particularly important to patients with hyperhidrosis (based on the previous qualitative study reported by Kamudoni et al. [13]) were retained. The last two showed optimal fit during the EFA. As previously noted, results from statistical models may not always address patient priorities, raising the need for their explicit consideration during item reduction [31]. The final 18 items were grouped under two domains, daily life activities domain (with 6 items) and psychosocial impact (with 12 items), supported by the results of the EFA (Fig. 3). The items were scored on a 3-point scale: no, not at all = 0; a little = 1; and very much = 2.

### Validation of the final HidroQoL

#### Reliability

The HidroQoL showed strong internal consistency (baseline responses, cohort 2, *n* = 260) [Cronbach’s alpha: overall scale, *α* = 0.89, daily life activities domain, *α* = 0.76, psychosocial domain, *α* = 0.86]. The corrected item–total correlation coefficients ranged from 0.376 to 0.618, reflecting a well-balanced scale, i.e. all items were tapping into the underlying construct. The time between the first (baseline) and the second (first follow-up) assessment was 7 days for the majority of patients (70 %) (max - 18 days, min - 5 days). The results of the test-retest reliability assessment demonstrated strong reproducibility of the HidroQoL scores [Intra-class correlation (95 % CI): overall scale, ICC = 0.93 (0.89, 0.95), *p* < 0.001; daily life activities, ICC = 0.88 (0.83, 0.92), *p* < 0.001; psychosocial impact, ICC = 0.914 (0.87, 0.94), *p* < 0.001]. Similar results were observed on the individual items (ICC range 0.792–0.876). Strong reliability was also shown in the USA and the UK subsamples separately (Online SM 5). These findings suggest that the HidroQoL can be reliably used for individual-level assessment of QoL such as in routine clinical practice.

#### Construct validity

All the hypotheses tested to assess construct validity of the HidroQoL were confirmed. The HidroQoL scores correlated moderately with the HDSS score [Spearman’s rank-sum correlation (*r*): overall score, *r* = 0.59, *p* < 0.001; daily life activities, *r* = 0.55, *p* < 0.001; psychosocial domain, *r* = 0.53, *p* < 0.001]. The general question (GQ) score was positively correlated with HidroQoL scores [overall score, *r* = 0.54, *p* < 0.001; daily life activities, *r* = 0.48, *p* < 0.001; psychosocial impact, *r* = 0.50, *p* < 0.001]. The DLQI’s total score had a positive correlation with the HidroQoL scores (overall scale, *r* = 0.60, *p* < 0.001, daily life activities domain, *r* = 0.52, *p* < 0.001; psychosocial impact, *r* = 0.56, *p* < 0.001). Finally, the HidroQoL scores correlated with the ‘amount of time spent in managing hyperhidrosis daily’ (*r* = 0.42, *p* < 0.001). Further, the Skindex-17’s symptom and psychosocial scales also showed slight-substantial correlations with the HidroQoL scores [overall score, *r* = 0.26, *r* = 0.63; daily life activities domain, *r* = 0.17, *r* = 0.48; psychosocial impact, *r* = 0.28, *r* = 0.63]. Construct validity was also established for the US and the UK patients, separately (Online SM 6). The HidroQoL therefore is capable of measuring the key impacts of disease central to hyperhidrosis especially those that are influenced by the severity of the condition.

#### Responsiveness

Patients were grouped according to change in their disease severity (HDSS score) from baseline to second follow-up assessment (based on patients completing second follow-up after 15–35 days, *n* = 90, cohort 2). Nineteen patients (21 %) had minimally improved (change in HDSS score, +1), 64 (72 %) had not changed and 6 patients (7 %) had minimally worsened. In the minimally improving group, the mean change scores were 1.05, 2.05 and 3.1, for the *daily life activities* (*p* = 0.09), *psychosocial domain* (*p* = 0.003) domains and the overall scale (*p* = 0.005), respectively. The change in the overall scale score corresponded to a Cohen’s effect sizes of 0.47 (95 % CI −0.24, 1.05). This indicates that the HidroQoL was sensitive to change in the patient’s condition. Further, the HidroQoL score differentiated between patients in the three groups of change [mean change, t_2_–t_1_: minimally improved, −3.1 ± 3.85; no change, −1.58 ± 4.49; minimally worsened, 3 ± 5.25, KW-test: overall scale, chi-squared = 6.9, *p* = 0.031; daily life activities domain, chi-squared statistic = 6.8, *p* = 0.034; and psychosocial domain, chi-squared = 5.9, *p* = 0.051)]. These results provide the evidence that the HidroQoL meets the critical requirements for measuring QoL in a longitudinal context, the ability to distinguish treatment responders in addition to sensitivity to change.

## Discussion

The impact of hyperhidrosis on the patient’s life is considerable [32]. HRQoL impacts of hyperhidrosis are known to be worse than in some skin conditions (such as psoriasis and atopic dermatitis [33]) or (chronic illnesses such as renal failure and diabetes [34]). The measurement of such impacts has, until now, been a challenge, partly due to use of inappropriate measures such as those assessing disease severity rather than impact on the patients’ lives and a lack of appropriately developed and validated measures of disease-specific impact. The current study describes the development and validation of a new hyperhidrosis-specific QoL instrument, the Hyperhidrosis Quality of Life Index (HidroQoL©).

The new instrument differs in emphasis and content coverage from current disease-specific measures. For example, the Hyperhidrosis Questionnaire [35] has five domains including a domain on symptoms ‘*physical domain.*’ Similarly, in the Hyperhidrosis Quality of Life questionnaire [36], one of its four domains seems to represent severity rather than impacts of disease. Symptom and severity-related items are not included in the new instrument (the HidroQoL), as these were demonstrated by factor analysis and Rasch analysis to lie outside the domain of QoL impact of hyperhidrosis. Further, impacts on self-image and embarrassment not included in the Hyperhidrosis Impact Questionnaire [37] are covered in the HidroQoL.

An online social networking patient population represents a number of advantages for the HidroQoL©. The measure’s content reflects the experiences of patients often excluded from PRO development, the non-clinic patients. In hyperhidrosis, this group makes up 65 % of all patients [3]. The participation of patients from multiple countries in the study enhanced the universality of the new measure. Furthermore, the involvement of patients as experts, evaluating the quality and relevance of the HidroQoL's  content, during the content validations step, contributed to the patient-centredness of the HidroQoL. On matters of item relevance, the views of the patient panel carried more weight as they were reporting based on first-hand experience. Input from the patient and clinician experts provided useful insights facilitating the revision of the measure.

The initial construct validation and item reduction were based on a large and heterogeneous patient population reflecting all forms of hyperhidrosis and different levels of disease severity. Use of techniques from modern test theory during this step means that the HidroQoL reflects the highest measurement standards and precision, e.g. invariance of items across various demographic groups. The item reduction based on exploratory factor analysis and Rasch analysis resulted in slightly different item selection, with 11 common items. The factor analysis–Rasch analysis friction can be traced to the lack of a ‘linear ruler’ (continuum) on which the items are ordered according to the level of impairment in the underlying QoL construct they refer to, within EFA [38]. Further, local dependence (influence of responses of one item on another) is explicitly addressed in the Rasch model. Nonetheless, the EFA was useful in identifying the domains of the measure. The HidroQoL's item scores can be summed to form sub- and overall scale scores: Q1–Q6 as the *daily life activities domain* score, items Q7–Q18 as the *psychosocial impact domain* score and all items as overall scale score. The individual items are assumed to have equal weighting, supporting a simple arithmetic summation in the calculation of domain and overall score. This is underpinned by properties of the Rasch model: (1) item responses are determined by the difference between the location of an item on the latent variable (i.e. level of QOL impairment being targeted by an item) and the location of the person on the same variable (i.e. level of QOL impairment of a person) [39]; (2) items are assumed to have equal ability to discriminate (slope parameter), while targeting different levels of the latent variable [40].

As item reduction based on statistical models depends on the pattern of responses or correlations among items, the preferences/priorities of the patient are not directly taken into account. This poses a risk to the content validity of a measure, if items core to the construct under assessment demonstrate poor fit and are consequently removed. Therefore, it is essential to make qualitative considerations when making final decisions related to item revision. To resolve this tension, for the HidroQoL, three items were retained to preserve the integrity of the construct (these emerged as the most prevalent themes during the qualitative interviews preceding the current work) [13].

The study design considered an interval of 7 days for the first follow-up assessment (to test reproducibility). Seven days are considered appropriate interval between assessment points (test 1 and 2) for such a psychometric property, as it is not too long for the patient’s condition to have changed and not too short to risk patients remembering responses to a previous assessment [41, 42] to avoid underestimation and overestimation. The planned duration of the second follow-up of 21 days for the assessment of responsiveness was based on expected time to euhidrosis following non-surgical treatment (excluding Botox), such as Aluminium Chloride (1–3 weeks) and Iontophoresis (1–4 weeks) [4]. During data collection, some patients responded to their first and second follow-up assessments earlier or later that instructed. Their observations were still included in the analysis.

The HidroQoL may be applied in routine clinical practice in various ways. First, scores for the different individual items might alert the clinicians regarding the area the patient might be experiencing the most problems, facilitating discussion between patient and clinicians during consultation [43]. Where necessary, the clinician may be able to refer the patient for psychiatric services, counselling or other services, based on results of the HidroQoL. Second, the strong association of the HidroQoL scores with patient’s disease severity (HDSS scores) suggests that the HidroQoL might also be useful in the diagnosis of hyperhidrosis [4]. For example, a score above a given cut-off value (to be determined in future studies) might be indicative of significant HRQoL impacts, which may serve as confirmation of a hyperhidrosis diagnosis, in addition to fulfilment of clinical criteria. This is particularly important considering the role the assessment of daily life impacts plays in the diagnosis and clinical management of hyperhidrosis [4].

Further, the established responsiveness and test–retest reliability of the measure means that the HidroQoL may be used for monitoring treatment response for patients. It is important to bear in mind that the currently presented psychometric properties are based on the online version of the HidroQoL. As this version was designed with minimal alterations to the paper and pencil version (e.g. use of radial buttons as opposed to check boxes for responses), a full validation study may not be necessary to confirm the observed psychometric attributes for the paper and pencil version of the HidroQoL (see Coons et al. [44]).

This study faced a number of limitations. First, not all patients from baseline assessment completed the consecutive follow-up assessments. It was not possible to ascertain the reasons for non-response, given the study design. Second, as the data collection was undertaken electronically with no clinic visits, there was no clinical confirmation of the participant’s diagnosis as hyperhidrosis. An idea during the design phases of the study was to request patients for records that would demonstrate their hyperhidrosis-diagnosis such as a prescription receipt. This was, however, not implemented considering the potential burden on the patients. Nonetheless, 85 % of patients self-reported seeing a clinician for the condition.

Further, online social networking patient populations may be associated with some self-selection bias. A previous study reported greater dissatisfaction with treatment and less self-rated therapeutic benefit in an online psoriatic patient population relative to a clinic population [45]. In addition, patients’ membership to online social networking communities presumes computer literacy and internet access, automatically excluding those without.

Nevertheless, a number of considerations exonerate the above concerns. First, it could be argued that the study sample is more representative of the hyperhidrosis patient population at large because it included both clinic and non-clinic patients. The current levels of internet usage (UK, 82 %; USA, 77.2 %) [46], also, suggest that those without access might actually be in the minority. Furthermore, whereas there might be practical and logistical challenges with obtaining sufficient patient numbers in local clinics (due to the prevalence of hyperhidrosis—2.8 %, the majority of whom do not seek for medical attention [3]), online patient support communities offer an alternative source of research participants, without geographical limitations. Pertinently, the current sample showed heterogeneity across important disease characteristics.

The current research sets a new standard for the measurement of HRQoL in hyperhidrosis. A ‘third-generation’ disease-specific QoL instrument for hyperhidrosis, rooted in the experiences of patients and validated in a large international sample based on modern test theory, is now available. The perennial nature of instrument validation means that there is still further work to be carried out on the new instrument. A study to identify minimal clinically important difference (MCID) and scale banding system for the HidroQoL scores has been planned. Even more importantly, the psychometric properties demonstrated by the HidroQoL will need to be confirmed in patients in clinic settings.

## Electronic supplementary material

Below is the link to the electronic supplementary material.

Scree plot showing optimal number of factors for the 21 items of the HidroQoL following item reduction using exploratory factor analysis. The optimal number of factors for extraction is identified by counting the factors lying to the left of the curve’s elbow. Factors to the right represent random rather than meaningful co-variation among the items (TIFF 17 kb)

Test characteristic curves of the HidroQoL total score and the latent QoL variable, by age groups. The relationship between the HidroQoL total raw score and the latent QoL variable was similar for the different age groups, indicating absence of bias for the total score in spite of DIF observed in some items (JPEG 28 kb)

Test characteristic curves of the HidroQoL total score and the latent QoL variable, by body area affected. The relationship between the HidroQoL total raw score and the latent QoL variable was similar for patients with different sites of hyperhidrosis (JPEG 39 kb)

Test characteristic curves of the HidroQoL total score and the latent QoL variable, by HDSS score (disease severity). The relationship between the HidroQoL total raw score and the latent QoL variable was similar for patients with different levels of disease severity (JPEG 31 kb)

Test characteristic curves of the HidroQoL total score and the latent QoL variable, by comorbidity. The relationship between the HidroQoL total raw score and the latent QoL variable was similar for patients with different levels of disease severity (JPEG 22 kb)

Supplementary material 6 (DOCX 19 kb)
