# Supplementary figures and images for: The development and validation of a disease-specific quality of life measure in hyperhidrosis: the Hyperhidrosis Quality of Life Index (HidroQOL©)
Source: Qual Life Res. 2014 Nov 1;24(4):1017–27. doi: 10.1007/s11136-014-0825-2 (PMC4366556; doi:10.1007/s11136-014-0825-2)

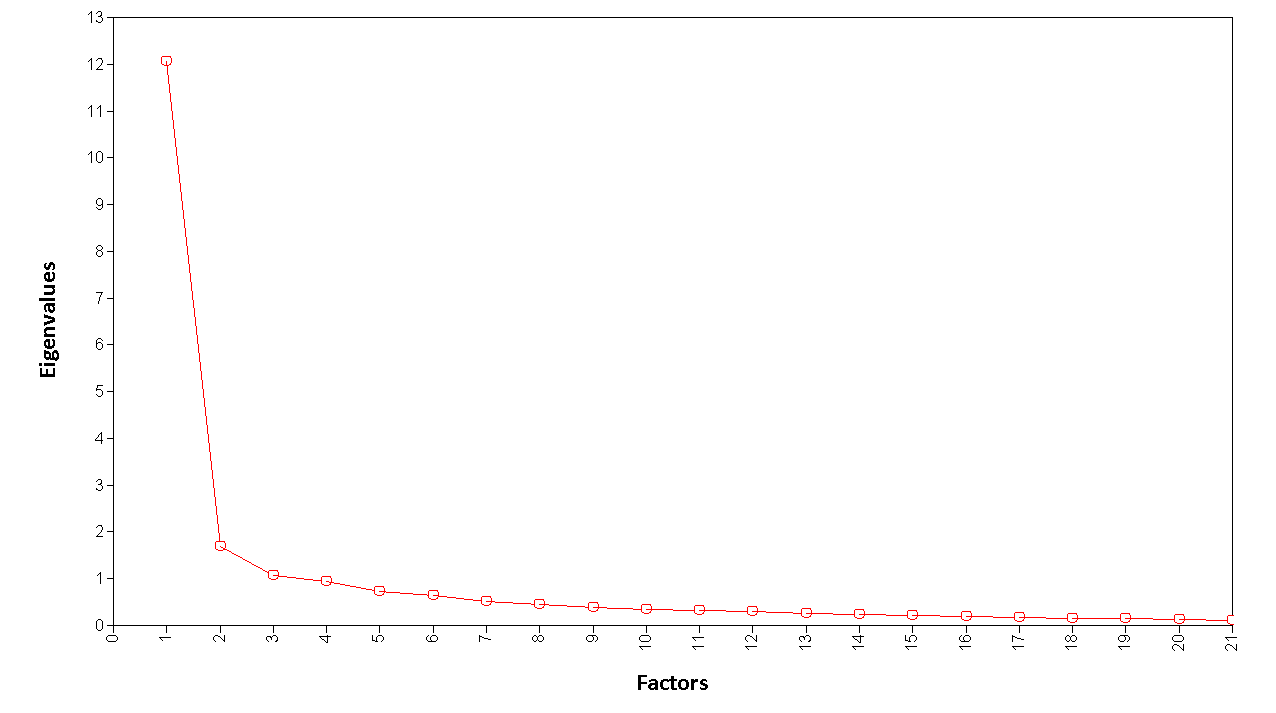

Supplement: Supplementary file 1 — Scree plot showing optimal number of factors for the 21 items of the HidroQoL following item reduction using exploratory factor analysis. The optimal number of factors for extraction is identified by counting the factors lying to the left of the curve’s elbow. Factors to the right represent random rather than meaningful co-variation among the items (TIFF 17 kb) [file 11136_2014_825_MOESM1_ESM.tif]

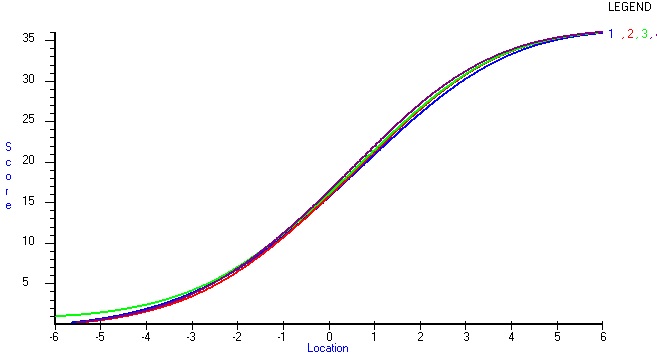

Supplement: Supplementary file 2 — Test characteristic curves of the HidroQoL total score and the latent QoL variable, by age groups. The relationship between the HidroQoL total raw score and the latent QoL variable was similar for the different age groups, indicating absence of bias for the total score in spite of DIF observed in some items (JPEG 28 kb) [file 11136_2014_825_MOESM2_ESM.jpg]

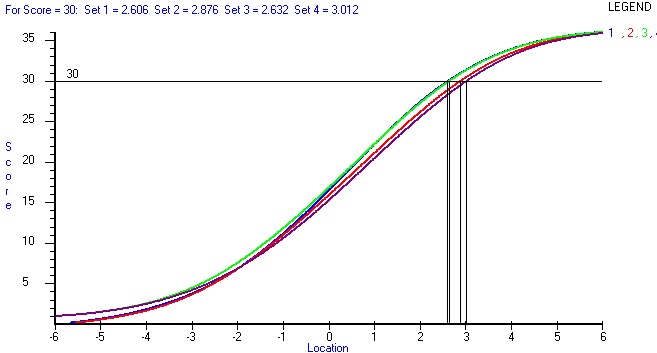

Supplement: Supplementary file 3 — Test characteristic curves of the HidroQoL total score and the latent QoL variable, by body area affected. The relationship between the HidroQoL total raw score and the latent QoL variable was similar for patients with different sites of hyperhidrosis (JPEG 39 kb) [file 11136_2014_825_MOESM3_ESM.jpg]

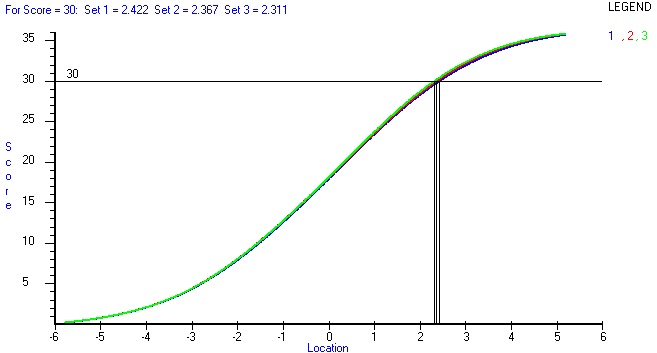

Supplement: Supplementary file 4 — Test characteristic curves of the HidroQoL total score and the latent QoL variable, by HDSS score (disease severity). The relationship between the HidroQoL total raw score and the latent QoL variable was similar for patients with different levels of disease severity (JPEG 31 kb) [file 11136_2014_825_MOESM4_ESM.jpg]

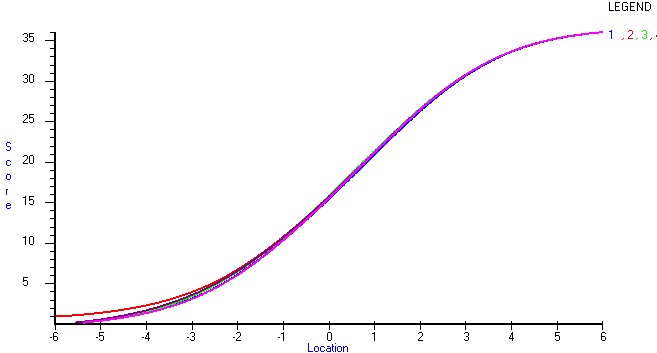

Supplement: Supplementary file 5 — Test characteristic curves of the HidroQoL total score and the latent QoL variable, by comorbidity. The relationship between the HidroQoL total raw score and the latent QoL variable was similar for patients with different levels of disease severity (JPEG 22 kb) [file 11136_2014_825_MOESM5_ESM.jpg]
