# Supplementary material for: The development and validation of a disease-specific quality of life measure in hyperhidrosis: the Hyperhidrosis Quality of Life Index (HidroQOL©)
Source: Qual Life Res. 2014 Nov 1;24(4):1017–27. doi: 10.1007/s11136-014-0825-2 (PMC4366556; doi:10.1007/s11136-014-0825-2)
Supplement: Supplementary file 6 — Supplementary material 6 (DOCX 19 kb) [file 11136_2014_825_MOESM6_ESM.docx]

**THE DEVELOPMENT AND VALIDATION OF A DISEASE-SPECIFIC QUALITY OF LIFE MEASURE IN HYPERHIDROSIS: THE HYPERHIDROSIS QUALITY OF LIFE INDEX (HidroQOL ©)**

**QUALITY OF LIFE RESERCH**

**Online Supplementary Material 5**

Kamudoni, P.^1^, Mueller, B.^2^ and Salek, M.S.^1^

1 Centre for Socioeconomic Research, School of Pharmacy and Pharmaceutical Sciences, Cardiff University, Redwood Building, Kind Edward VII Avenue, Cardiff CF 10 3NB, UK.

2. Medical Science and Operations Department, Riemser GmbH, Greifswald, Germany.

Corresponding author details:

Prof Sam Salek

Centre for Socioeconomic Research,

School of Pharmacy and Pharmaceutical Sciences,

Cardiff University,

Redwood Building,

King Edward VII Avenue,

Cardiff CF 10 3NB,

UK.

Tel: +44(0) 2920876017

Email: salekss@cf.ac.uk

**Table 1: Internal consistency of the HidroQoL**

| **HidroQoL scale** | **Cronbach’s alpha coefficient** | | |
| --- | --- | --- | --- |
|  | Pooled† (n = 260) | USA (n =142) | UK (n =73) |
| *Overall scale* | 0.89 | 0.89 | 0.89 |
| *DLA* | 0.76 | 0.78 | 0.72 |
| *PS* | 0.86 | 0.87 | 0.85 |

**Notes:**

1*. DLA*, Daily life activities domain. *PS*, Psychosocial life domain

2. † The pooled sample included patients from 20 countries including the USA and UK

**Table 2: Test-retest reliability of the HidroQoL**

| **HidroQoL scale** | **Intra-class correlation coefficient (95% CI)** | | |
| --- | --- | --- | --- |
|  | Pooled† (n = 104) | USA (n = 64) | UK (n =22) |
| *Overall scale* | 0.926**(0.885, 0.952) | 0.919** (0.868, 0.951) | 0.932**(0.74, 0.976) |
| *DLA* | 0.88**(0.828, 0.921) | 0.892**(0.824, 0.934) | 0.866**(0.689, 0.943) |
| *PS* | 0.914**(0.868, 0.943) | 0.904**(0.843, 0.941) | 0.919**(0.649, 0.973) |

**Notes:**

1. *DLA*, Daily life activities domain. *PS*, Psychosocial life domain

2. † The pooled sample included patients from 20 countries including the USA and UK

3. **, p > 0.001, *, p > 0.05.

**Table 3: Convergence of the HidroQoL scores to related measures^¥^**

| **HidroQoL scale** | **External variable** | **Correlation coefficient** | | |
| --- | --- | --- | --- | --- |
|  |  | *Pooled*  (n = 260) | *USA*  (n = 64) | *UK*  (n =73) |
| *Overall scale* | *Skindex-sy* | 0.264** | 0.259* | 0.366* |
| *DLA* | *Skindex-sy* | 0.172* | 0.193* | 0.302* |
| *PS* | *Skindex-sy* | 0.284** | 0.261* | 0.380** |
| *Overall scale* | *Skindex-ps* | 0.627** | 0.591** | 0.706** |
| *DLA* | *Skindex-ps* | 0.482** | 0.473** | 0.525** |
| *PS* | *Skindex-ps* | 0.623** | 0.553** | 0.729** |
| *Overall scale* | *DLQI total score* | 0.598^**^ | 0.562^**^ | 0.659^**^ |
| *DLA* | *DLQI total score* | 0.519^**^ | 0.502^**^ | 0.589^**^ |
| *PS* | *DLQI total score* | 0.556^**^ | 0.500^**^ | 0.633^**^ |
| *Overall scale* | *DMT* | 0.420^**^ | 0.432^**^ | 0.452^**^ |
| *DLA* | *DMT* | 0.450^**^ | 0.544^**^ | 0.423^**^ |
| *PS* | *DMT* | 0.364^**^ | 0.336^**^ | 0.406^**^ |
| *Overall scale* | *GQ* | 0.536^**^ | 0.592^**^ | 0.502^**^ |
| *DLA* | *GQ* | 0.476^**^ | 0.568^**^ | 0.446^**^ |
| *PS* | *GQ* | 0.503^**^ | 0.516^**^ | 0.515^**^ |
| *Overall scale* | *HDSS* | 0.589^**^ | 0.641^**^ | 0.530^**^ |
| *DLA* | *HDSS* | 0.550^**^ | 0.645^**^ | 0.497^**^ |
| *PS* | *HDSS* | 0.529^**^ | 0.539^**^ | 0.513^**^ |

**Note:**

1. *DLA*, Daily life activities domain. *PS*, Psychosocial life domain. *Skindex-sy*, Skindex-17 Symptom subscale. *Skindex-ps*, Skindex-17 psychosocial domain. *DLQI,* Dermatology Life Quality Index. *DMT*, Daily time spent on managing hyperhidrosis. *GQ*, General Question (on overall impact of hyperhidrosis on patient). *HDSS*, Hyperhidrosis Disease Severity Scale.
2. ¥, spearman rank sum correlation.
3. **, p > 0.001, *, p > 0.05.
4. †, the pooled sample included patients from 20 countries including the USA and UK
